# CD8^+^ T-cell specificity is compromised at a defined MHCI/CD8 affinity threshold

**DOI:** 10.1038/icb.2016.85

**Published:** 2016-11-08

**Authors:** Tamsin Dockree, Christopher J Holland, Mathew Clement, Kristin Ladell, James E McLaren, Hugo A van den Berg, Emma Gostick, Kelly L Miners, Sian Llewellyn-Lacey, John S Bridgeman, Stephen Man, Mick Bailey, Scott R Burrows, David A Price, Linda Wooldridge

**Affiliations:** 1 Institute of Infection and Immunity, Cardiff University School of Medicine, Cardiff, UK; 2 Faculty of Health Sciences, University of Bristol, Bristol, UK; 3 Mathematics Institute, University of Warwick, Coventry, UK; 4 Institute of Cancer and Genetics, Cardiff University School of Medicine, Cardiff, UK; 5 Cellular Immunology Laboratory, QIMR Berghofer Medical Research Institute, Brisbane, QLD, Australia; 6 Human Immunology Section, Vaccine Research Center, National Institute of Allergy and Infectious Diseases, National Institutes of Health, Bethesda, MD, USA

## Abstract

The CD8 co-receptor engages peptide-major histocompatibility complex class I (pMHCI) molecules at a largely invariant site distinct from the T-cell receptor (TCR)-binding platform and enhances the sensitivity of antigen-driven activation to promote effective CD8^+^ T-cell immunity. A small increase in the strength of the pMHCI/CD8 interaction (~1.5-fold) can disproportionately amplify this effect, boosting antigen sensitivity by up to two orders of magnitude. However, recognition specificity is lost altogether with more substantial increases in pMHCI/CD8 affinity (~10-fold). In this study, we used a panel of MHCI mutants with altered CD8-binding properties to show that TCR-mediated antigen specificity is delimited by a pMHCI/CD8 affinity threshold. Our findings suggest that CD8 can be engineered within certain biophysical parameters to enhance the therapeutic efficacy of adoptive T-cell transfer irrespective of antigen specificity.

CD8^+^ T cells recognize antigens in the form of short peptide fragments bound to major histocompatibility complex class I (MHCI) molecules on the target cell surface.^1^ Specific engagement of peptide-MHCI (pMHCI) complexes via the clonotypically expressed αβ T-cell receptor (TCR) triggers a range of effector functions that play a critical role in protective immunity against intracellular infections and various malignancies. The ability to identify and eliminate cancerous cells *in vivo* is particularly intriguing^2, 3^ and promises novel therapies based on the immunobiology of CD8^+^ T cells. Indeed, adoptive transfer of *in vitro*-expanded CD8^+^ T cells can cause tumour regression in the clinical setting.^4, 5^ These seminal observations have sparked great interest in the use of cellular therapy to combat cancer.^6, 7^ However, a number of obstacles preclude the widespread use of this approach. In biological terms, one key limitation relates to the naturally low affinity of self-derived antigen-specific TCRs,^8, 9^ which constrains the functional properties of tumour-associated antigen-specific CD8^+^ T-cell populations. This intrinsic problem stems from the negative selection of high-affinity autoreactive αβ TCR clonotypes during thymic education and most likely explains why it has proven difficult to develop cancer vaccines in the absence of a clear oncogenic microbial agent. Although high-affinity TCRs can be engineered to circumvent suboptimal antigen recognition, most notably via phage display technology,^10, 11^ the requirement to reiterate this process for each pMHCI specificity tailored to individual tumour proteomes is a major barrier to therapeutic applicability.

The surface-expressed CD8αβ glycoprotein (CD8 from here on) serves as a co-receptor for MHCI-restricted T cells.^12^ CD8 binds to a largely invariant region of MHCI at a site distinct from the TCR-binding platform and acts to enhance T-cell antigen sensitivity by up to six orders of magnitude.^12, 13, 14^ This effect is mediated via several mechanisms, including: (i) promotion and stabilization of the TCR/pMHCI interaction at the cell surface;^15, 16, 17, 18^ (ii) recruitment of signalling molecules to the intracellular side of the TCR/CD3ζ complex;^19, 20, 21, 22^ and (iii) localization of TCR/pMHCI complexes within specialized membrane microdomains enriched for early intracellular signal transduction molecules.^23, 24^ These properties can potentially be harnessed to modulate antigen-specific CD8^+^ T-cell immunity. It is notable in this regard that pMHCI/CD8 binding is characterized by very low solution affinities (average *K*_D_~145 μM).^25^ Moreover, an incremental increase in the strength of this interaction (*K*_D_~98 μM) can boost antigen sensitivity by up to 100-fold.^17, 26^ Such manipulations are globally applicable across TCR specificities due to the non-polymorphic nature of CD8, thereby providing a generic opportunity to enhance CD8^+^ T-cell reactivity for therapeutic purposes.^27^ However, substantial increases in pMHCI/CD8 affinity can abrogate antigen specificity.^28^

In this study, we used a panel of MHCI mutants with altered CD8-binding properties to show that the specificity of peptide-dependent TCR recognition is maintained within a defined pMHCI/CD8 affinity window. Collectively, the data provide biophysical guidelines for the rational design of high-affinity CD8 molecules to optimize the therapeutic efficacy of adoptive T-cell transfer.

## Results

### Development of a novel MHCI mutant to probe the pMHCI/CD8 interaction

The pMHCI/CD8 interaction is characterized by very low solution binding affinities and extremely rapid kinetics.^29, 30, 31^ Although some variation exists between different MHCI molecules due to polymorphisms that affect the CD8 binding site, the average pMHCI/CD8 interaction occurs with an equilibrium dissociation constant (*K*_D_) ~145 μM (range=100–220 μM).^25, 32^ Substantially weaker pMHCI/CD8 solution binding affinities have been reported for human leukocyte antigen (HLA) A*6801, HLA B*4801 and HLA B*8101.^22, 25^ The introduction of a glutamine (Q) to glutamic acid (E) substitution at position 115 of the MHCI α2 domain increases the pMHCI/CD8 interaction by ~1.5-fold (*K*_D_~98 μM) without impacting the TCR/pMHCI binding platform.^26^ This mutation significantly enhances the sensitivity of pMHCI antigen recognition (up to 100-fold) without compromising TCR-mediated specificity. In contrast, a human to murine MHCI α3 domain switch increases the pMHCI/CD8 interaction by ~15-fold (*K*_D_~11 μM) and bypasses the requirement for cognate TCR engagement.^28^

**Figure 1:**
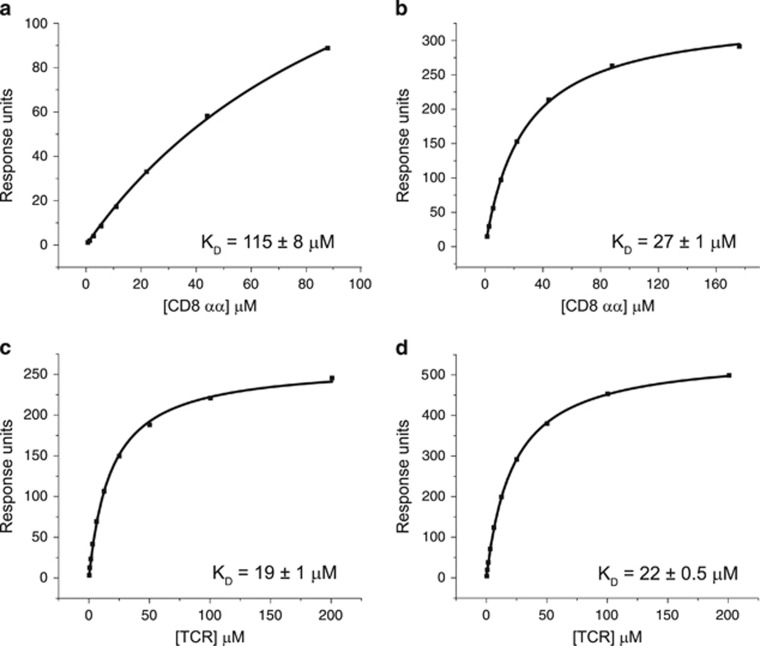
A2/K^b^ A245V exhibits enhanced affinity for CD8 without impacting the TCR/pMHCI interaction. Biotinylated A2 (**a**, **c**) or A2/K^b^ A245V (**b**, **d**) monomers refolded with wild type β_2_m and the heteroclitic peptide ELAGIGILTV were immobilized on a streptavidin-coated BIAcore chip. Serial dilutions of soluble human CD8αα (**a**, **b**) or MEL5 TCR (**c**, **d**) were flowed over the chip to measure equilibrium binding by surface plasmon resonance. Data were analyzed using BIAevaluation 3.1, Microsoft Excel and Origin 6.1.

**Figure 2:**
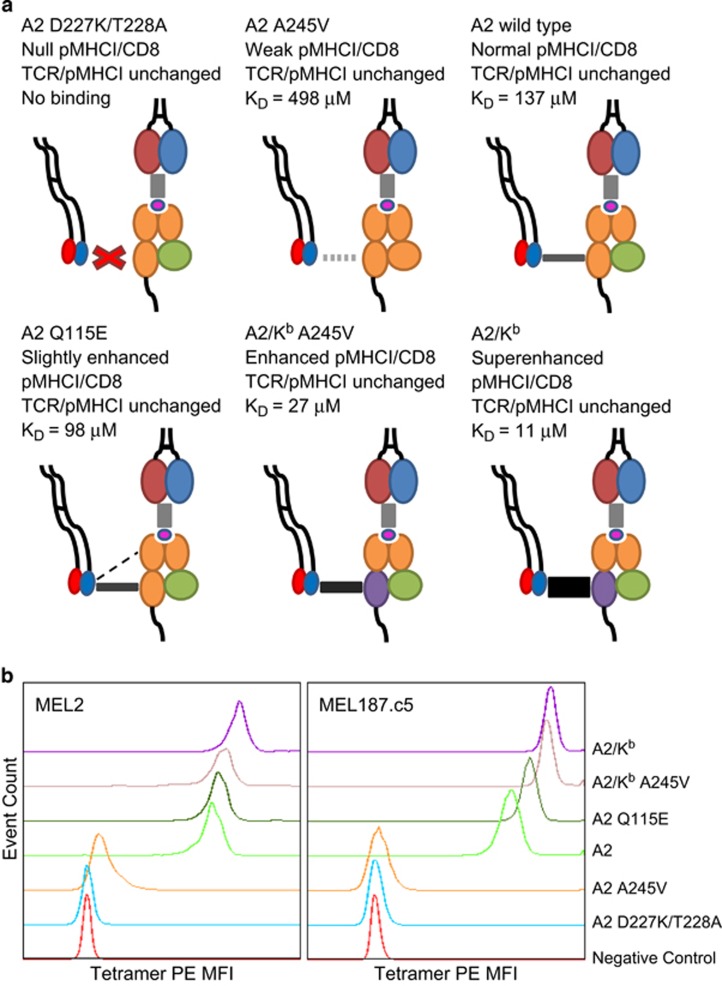
Increasing the strength of the pMHCI/CD8 interaction enhances pMHCI binding at the cell surface. (**a**) Schematic representation of the six different MHCI mutants spanning a range of pMHCI/CD8 interaction affinities. None of the introduced mutations affect TCR/pMHCI binding. (**b**) 5 × 10^4^ clonal MEL2 or MEL187.c5 CD8^+^ T cells were stained with ViViD and the indicated ELAGIGILTV tetramer (A2 D227K/T228A, A2 A245V, A2, A2 Q115E, A2/K^b^ A245V or A2/K^b^) at 25 μg ml^−1^. Viable events are shown in concatenated histogram plots. Data were acquired using a FACSCantoII flow cytometer and analyzed with FlowJo software version 10.6.

To determine the pMHCI/CD8 affinity at which antigen specificity is lost, we introduced an alanine (A) to valine (V) substitution at position 245 of A2/K^b^ (a fusion molecule comprising the α1/α2 peptide-binding platform of HLA A*0201 and the α3 domain of H2-K^b^) to generate the novel MHCI mutant A2/K^b^ A245V. Surface plasmon resonance analysis revealed that A2/K^b^ A245V binds CD8 with a *K*_D_ of 27 μM (Figures 1a and b), while the TCR/pMHCI interaction remains unchanged (Figures 1c and d). Combined with previously developed mutants, we then had an extended panel for functional analysis that incorporated MHCI molecules spanning a range of CD8 interaction affinities as follows: abrogated (A2 D227K/T228A);^21^ weak (A2 A245V);^22^ wild type (A2); slightly enhanced (A2 Q115E);^26^ enhanced (A2/K^b^ A245V); and superenhanced (A2/K^b^).^28^ Importantly, none of these mutations affect the integrity of TCR binding to pMHCI (Table 1; Figure 2a).

### Increasing the strength of the pMHCI/CD8 interaction enhances pMHCI engagement at the cell surface

To investigate the relationship between pMHCI/CD8 affinity and pMHCI engagement at the cell surface, we generated fluorescent tetrameric complexes of A2 D227K/T228A, A2 A245V, A2, A2 Q115E, A2/K^b^ A245V and A2/K^b^ refolded with wild type β_2_ microglobulin (β_2_m) and the decamer peptide ELAGIGILTV, which is a heteroclitic variant of the Melan-A_26-35_ epitope EAAGIGILTV. These pMHCI tetramers were used at standardized concentrations to stain two different ELAGIGILTV-specific CD8^+^ T-cell clones (MEL2 and MEL187.c5). Tetramer staining of MEL2 and MEL187.c5 was very poor in the absence of an interaction with CD8 (A2 D227K/T228A) (Figure 2b). As the strength of the pMHCI/CD8 interaction increased, however, progressive increments in pMHCI tetramer staining were observed for both CD8^+^ T-cell clones. Thus, pMHCI engagement at the cell surface is enhanced in the presence of stronger pMHCI/CD8 interactions.

### pMHCI binding specificity is compromised at a defined pMHCI/CD8 affinity threshold

Standard wild type pMHCI tetramers bind cell surface TCRs with exquisite specificity.^33, 34^ In contrast, nonspecific binding occurs in the presence of a superenhanced pMHCI/CD8 interaction (*K*_D_~11 μM).^28^ To define the pMHCI/CD8 affinity threshold at which pMHCI binding specificity is compromised, we stained healthy donor peripheral blood mononuclear cells (PBMCs) with fluorescent tetrameric complexes of A2 D227K/T228A, A2 A245V, A2, A2 Q115E, A2/K^b^ A245V and A2/K^b^ refolded with wild type β_2_m and ELAGIGILTV.

**Figure 3:**
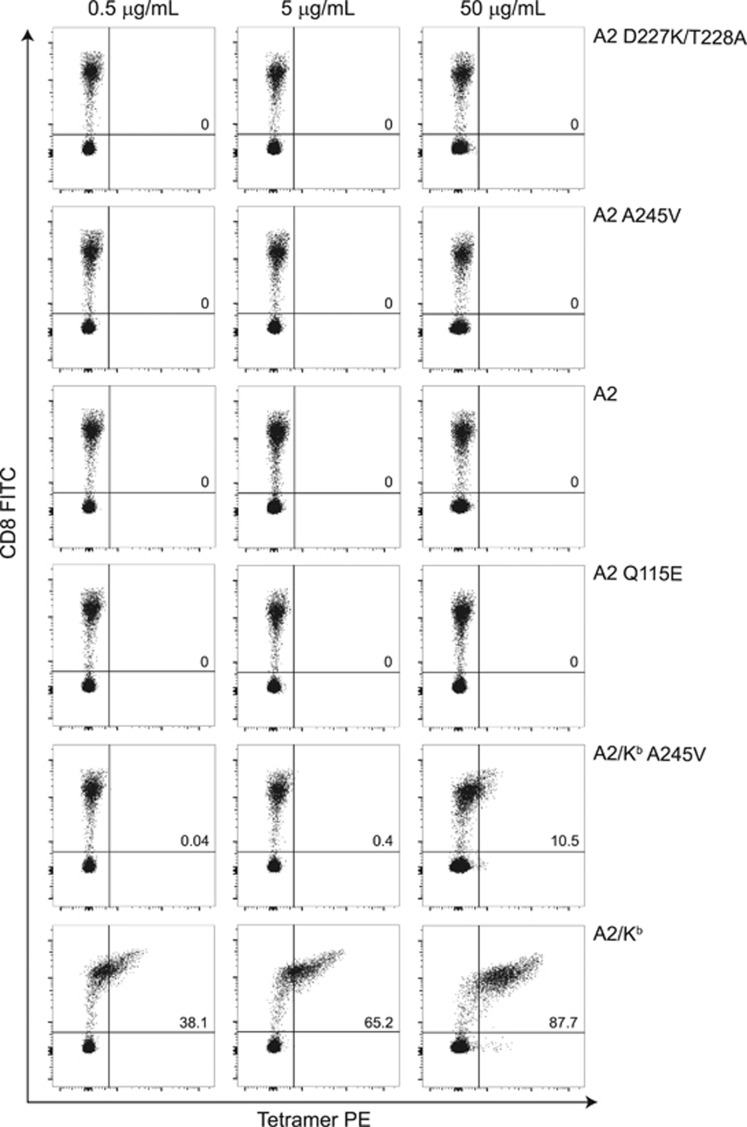
pMHCI binding specificity is compromised at a defined pMHCI/CD8 affinity threshold in A2^–^ donors. 1 × 10^6^ A2^–^ PBMCs were stained with ViViD and the indicated ELAGIGILTV tetramer (A2 D227K/T228A, A2 A245V, A2, A2 Q115E, A2/K^b^ A245V or A2/K^b^) at 0.5, 5 or 50 μg ml^−1^, followed by a panel of lineage-specific monoclonal antibodies as described in the Methods section. Plots are gated on live, CD3^+^ populations. Data were acquired using a FACSCantoII flow cytometer and analyzed with FlowJo software version 10.6. Values shown in the upper right quadrant indicate % tetramer^+^ CD8^+^ T cells.

First, we stained A2^–^ PBMCs. In the absence of alloreactivity, we would not expect these samples to harbour TCRs that recognize peptides in the context of A2. Any observable tetramer staining under these circumstances can therefore be attributed to peptide-independent recognition of pMHCI. No background staining was detected when A2^–^ PBMCs were stained with the A2 D227K/T228A, A2 A245V, A2 or A2 Q115E tetramers up to a concentration of 50 μg ml^−1^ (Figure 3). A similar pattern was observed with the A2/K^b^ A245V tetramer at 0.5 and 5 μg ml^−1^. In line with a concentration-dependent effect, however, the same reagent displayed moderate background staining at 50 μg ml^−1^. The A2/K^b^ tetramer was almost entirely nonspecific, as described in a previous report.^28^

**Figure 4:**
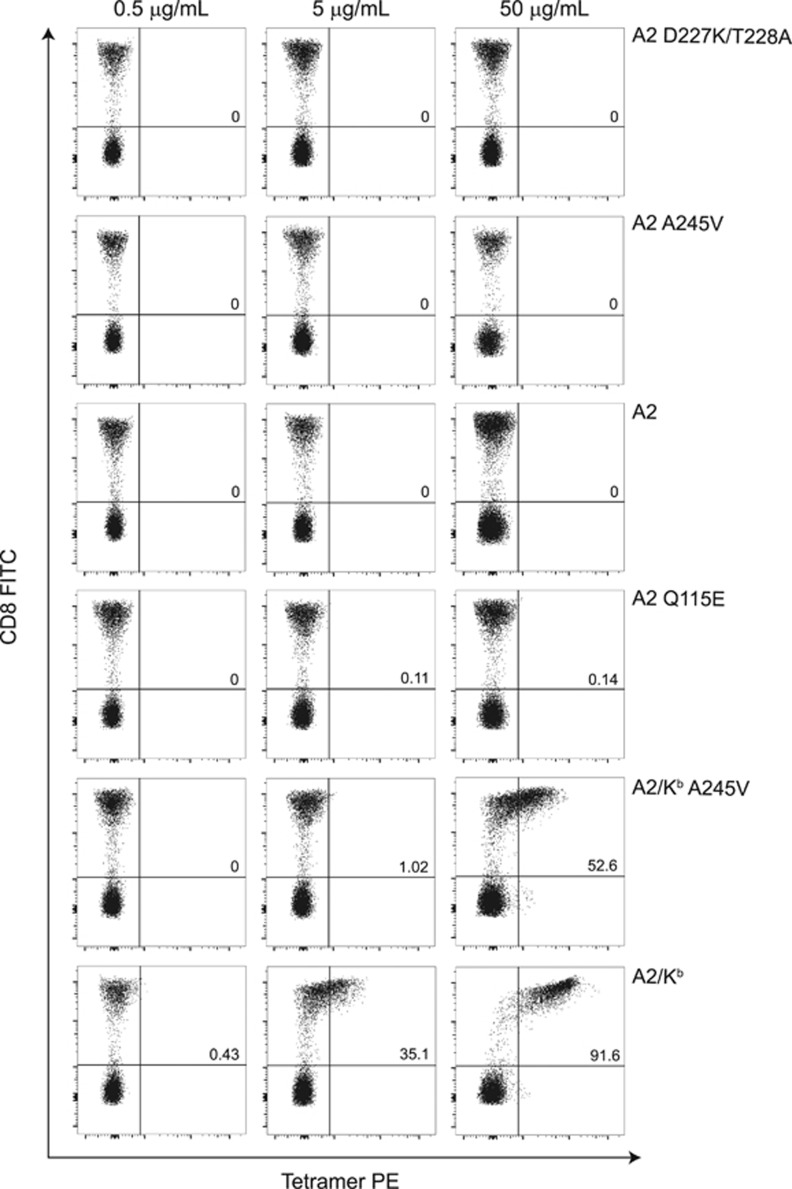
pMHCI binding specificity is compromised at a defined pMHCI/CD8 affinity threshold in A2^+^ donors. 1 × 10^6^ A2^+^ PBMCs were stained and analyzed as described in the legend for Figure 3. Values shown in the upper right quadrant indicate % tetramer^+^ CD8^+^ T cells.

Next, we repeated this analysis using A2^+^ PBMCs, which frequently harbour TCRs specific for ELAGIGILTV. The clonotypic repertoire in these samples is also shaped by positive selection to ensure an intrinsic level of reactivity with A2. Staining specificity was maintained with the A2 D227K/T228A, A2 A245V, A2 and A2 Q115E tetramers up to a concentration of 50 μg ml^−1^ (Figure 4). Similarly, no background staining was detected with the A2/K^b^ A245V tetramer at 0.5 and 5 μg ml^−1^. Reactivity was apparent with the same reagent at 50 μg ml^−1^, however, exceeding the levels observed in comparable experiments with A2^−^ PBMCs. The A2/K^b^ tetramer was again largely nonspecific, although this effect was not obvious at 0.5 μg ml^−1^.

**Figure 5:**
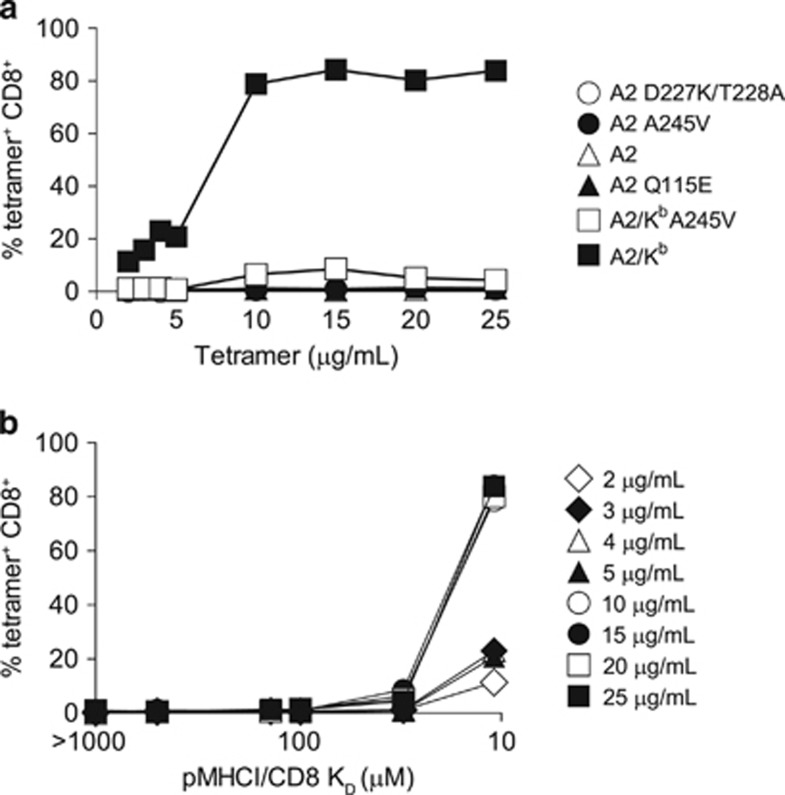
Detailed analysis of pMHCI binding specificity across a range of pMHCI/CD8 affinities in an A2^+^ donor. (**a**) 1 × 10^6^ A2^+^ PBMCs were stained and analyzed as described in the legend for Figure 3, with the exception that each tetramer was used at 2, 3, 4, 5, 10, 15, 20 or 25 μg ml^−1^. (**b**) The same data shown as % tetramer^+^ CD8^+^ T cells versus pMHCI/CD8 affinity.

**Figure 6:**
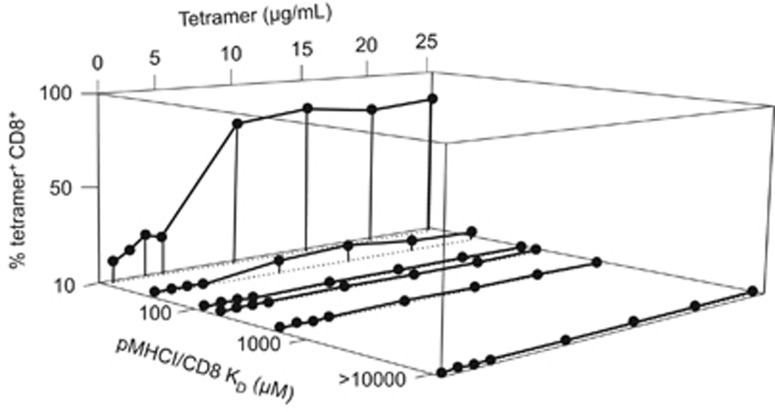
pMHCI binding specificity is a function of tetramer concentration and pMHCI/CD8 affinity. The percentage of tetramer^+^ CD8^+^ T cells varies with tetramer concentration (*P*=4.4 × 10^–3^; Friedman test). Modest to strong evidence was found for individual MHCI mutants (A2 D227K/T228A: *P*=1.6 × 10^–2^; A2 A245V: *P*=1.4 × 10^–1^; A2: *P*=1.4 × 10^–1^; A2 Q115E: *P*=1 × 10^–2^; A2/K^b^ A245V: *P*=5.4 × 10^–2^; A2/K^b^: *P*=8.8 × 10^–4^; Jonckheere–Terpstra test for increasing dependence on tetramer concentration). There was strong evidence for an effect of pMHCI/CD8 affinity on tetramer staining (*P*=3 × 10^–7^; Friedman test), although this was not apparent when data for the two lowest *K*_D_ values were excluded (*P*=1.7 × 10^–1^; Friedman test). Tetramer staining was strongly dependent on the *K*_D_ of the pMHCI/CD8 interaction (*P*<10^–7^; Jonckheere–Terpstra test for increasing dependence on *K*_D_). The virtual absence of staining at pMHCI/CD8 affinities >27 μM suggests that a value within this order of magnitude behaves as a threshold.

To consolidate these findings, we performed analogous experiments across a broader range of tetramer concentrations using PBMCs from a different A2^+^ donor (Figure 5a). Again, no loss of specificity was detected with the A2 D227K/T228A, A2 A245V, A2 or A2 Q115E tetramers up to a concentration of 25 μg ml^−1^. The A2/K^b^ A245V tetramer was also highly specific at ⩽5 μg ml^−1^, but modest reactivity was observed with the same reagent at>5 μg ml^−1^. Considerable background staining was apparent with the A2/K^b^ tetramer. To clarify these data, we plotted nonspecific staining as a function of tetramer concentration versus pMHCI/CD8 affinity (Figure 5b) and used non-parametric tests to examine the impact of these variables on tetramer binding at the cell surface (Figure 6). Our analyses revealed that loss of tetramer specificity does not occur gradually with incremental increases in the strength of the pMHCI/CD8 interaction. Instead, the specificity of pMHCI engagement is compromised beyond a certain pMHCI/CD8 affinity threshold, epitomized by the A2/K^b^ A245V (*K*_D_~27 μM) and A2/K^b^ (*K*_D_~11 μM) tetramers.

### T-cell activation specificity is compromised at a defined pMHCI/CD8 affinity threshold

**Figure 7:**
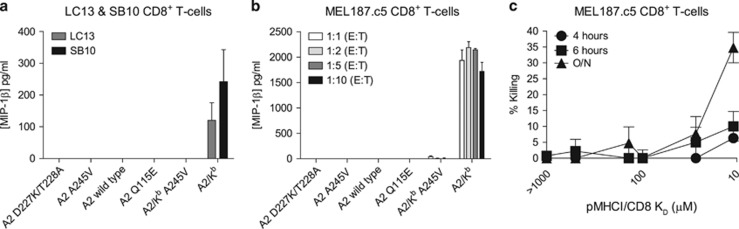
CD8^+^ T-cell activation specificity is compromised at a defined pMHCI/CD8 affinity threshold. (**a**) 3 × 10^4^ clonal SB10 or LC13 CD8^+^ T cells were incubated overnight with 6 × 10^4^ C1R B cells expressing A2 D227K/T228A, A2 A245V, A2, A2 Q115E, A2/K^b^ A245V or A2/K^b^. Supernatants were collected and assayed for macrophage inflammatory protein (MIP)-1β by enzyme-linked immunosorbent assay (ELISA). Data are shown corrected for background production of MIP-1β. (**b**) 3 × 10^4^ clonal MEL187.c5 CD8^+^ T cells were incubated overnight at the indicated E:T ratios with C1R B cells expressing A2 D227K/T228A, A2 A245V, A2, A2 Q115E, A2/K^b^ A245V or A2/K^b^. Supernatants were collected and assayed for macrophage inflammatory protein (MIP)-1β by ELISA. Data are shown corrected for background production of MIP-1β. (**c**) 1 × 10^4^ clonal MEL187.c5 CD8^+^ T cells were incubated with 2 × 10^3^ C1R B cells expressing A2 D227K/T228A, A2 A245V, A2, A2 Q115E, A2/K^b^ A245V or A2/K^b^ in standard chromium release assays as described in the Methods section. Data are shown as % killing versus pMHCI/CD8 affinity. Error bars represent s.d.

CD8^+^ T-cell activation is exquisitely sensitive, requiring <10 pMHCI molecules for full calcium release and mature synapse formation.^35^ As a consequence, effector functions can be elicited at cognate pMHCI concentrations well below those necessary for detectable tetramer binding.^36^ To determine the pMHCI/CD8 affinity at which activation specificity is lost, we used a panel of Hmy.2 C1R (C1R) B cells transduced to express A2 D227K/T228A, A2 A245V, A2, A2 Q115E, A2/K^b^ A245V or A2/K^b^ at equivalent surface densities. Nonspecific activation as a function of pMHCI/CD8 affinity was initially tested using the LC13 and SB10 CD8^+^ T-cell clones, which are neither restricted by nor alloreactive against A2.^37, 38^ After overnight stimulation, nonspecific macrophage inflammatory protein-1β release was only observed in the presence of A2/K^b^ C1R B cells (Figure 7a). Similar results were obtained with the A2-restricted CD8^+^ T-cell clone MEL187.c5 (Figure 7b).

To confirm these findings with a different effector read-out, we used the same panel of C1R B cells in standard chromium release assays with the MEL187.c5 CD8^+^ T-cell clone to measure peptide-independent cytotoxicity (Figure 7c). The A2 D227K/T228A, A2 A245V, A2 and A2 Q115E C1R B-cell targets remained largely intact throughout the experiment. Similarly, there was no detectable short-term killing of A2/K^b^ A245V C1R B cells. Marginal nonspecific lysis was apparent with the same targets after prolonged incubation, however, consistent with a subtle time-dependent effect triggering the release of cytolytic enzymes. The A2/K^b^ C1R B-cell targets were killed in substantial numbers over time. Collectively, these data mirror the corresponding tetramer staining patterns and indicate that CD8^+^ T-cell activation specificity is maintained below a defined pMHCI/CD8 affinity threshold (*K*_D_~27 μM).

## Discussion

Despite an extremely weak interaction with MHCI (average *K*_D_~145 μM), the CD8 co-receptor mediates profound biological effects that enhance the sensitivity of TCR-driven activation in response to cognate antigen.^12, 39^ A small increment in pMHCI/CD8 affinity can further amplify the functional consequences of this interaction, increasing antigen sensitivity in responding CD8^+^ T cells by up to 100-fold.^26^ These observations suggest a possible translational role for affinity-enhanced CD8 molecules.^27^ For example, the introduction of such modified co-receptors together with tumour-specific TCRs may facilitate the activation of engineered T cells in the presence of naturally expressed cancer antigens, compensating both for low-affinity TCR/pMHCI interactions and low-density cognate pMHCI expression on the target cell surface. However, excessive increases in the strength of the pMHCI/CD8 interaction (*K*_D_~11 μM) lead to nonspecific T-cell activation.^28^ It is therefore important to define the optimal affinity at which CD8 co-receptor engagement enhances pMHCI recognition without compromising the specificity of antigen-specific CD8^+^ T cells.

In this study, we used a panel of MHCI molecules spanning a range of CD8-binding affinities to delineate the impact of variable pMHCI/CD8 interactions on the specificity of TCR-mediated antigen recognition. Surface plasmon resonance studies confirmed that none of these mutations affect the TCR/pMHCI-binding platform. Tetrameric pMHCI complex engagement at the cell surface was enhanced in a stepwise manner with increasing pMHCI/CD8 affinities. In contrast, the specificity of pMHCI binding and T-cell activation was compromised at a defined pMHCI/CD8 affinity threshold (*K*_D_~27 μM).

Biophysical studies have shown that the murine pMHCI/CD8 interaction (average *K*_D_~49 μM) is considerably stronger than the human pMHCI/CD8 interaction (average *K*_D_~145 μM).^21, 25^ This peculiar feature of mice may act to enhance T-cell cross-reactivity, allowing a size-limited repertoire to provide effective coverage against a common universe of pMHCI antigens.^40^ It is also notable that the affinity of the murine pMHCI/CD8 interaction lies just below the specificity threshold defined in this study (*K*_D_~27 μM). A conserved optimum may therefore dictate the evolutionary limits of co-receptor binding within a functional mammalian immune system.

The data presented here suggest the existence of an affinity window that potentially enables optimization of the pMHCI/CD8 interaction for therapeutic purposes without nonspecific T-cell activation. However, it is important to note that CD8^+^ T cells are naturally cross-reactive and that this phenomenon is controlled to some extent by the CD8 co-receptor.^41, 42, 43^ It will therefore be important to examine this effect in more detail to avoid potentially dangerous off-target reactivity.^44, 45^ Nonetheless, the maintenance of CD8^+^ T-cell specificity below a supranormal pMHCI/CD8 affinity threshold offers an exciting opportunity to enhance the therapeutic efficacy of adoptive cell transfer irrespective of antigen specificity.

## Methods

### Cells

The following CD8^+^ T-cell clones were used in this study: (i) MEL2 and MEL187.c5, specific for the Melan-A-derived epitope ELAGIGILTV (residues 26–35) restricted by HLA A*0201 (A2); (ii) LC13, specific for the Epstein–Barr virus EBNA3A-derived epitope FLRGRAYGL (residues 339–347) restricted by HLA B*0801;^37^ and (iii) SB10, specific for the cytomegalovirus pp65-derived epitope CPSQEPMSIYVY (residues 103–114) restricted by HLA B*3508.^38^ Clones were maintained in RPMI 1640 containing 100 U ml^−1^ penicillin, 100 mg ml^−1^ streptomycin, 2 mM L-glutamine and 10% heat-inactivated fetal calf serum (R10; all components from Life Technologies, Carlsbad, CA, USA), supplemented with 2.5% Cellkines (Helvetica Healthcare, Geneva, Switzerland), 200 IU ml^−1^ interleukin-2 and 25 ng ml^−1^ interleukin-15 (both PeproTech, Rocky Hill, NJ, USA). Healthy donor PBMCs were isolated by standard density gradient centrifugation using Ficoll-Hypaque (GE Healthcare, Chicago, IL, USA). C1R B cells expressing full-length A2 and variants thereof were generated and maintained as described previously.^26^

### pMHCI tetramer staining and flow cytometry

Soluble pMHCI tetramers were produced as described previously.^17^ For A2 typing, 1 × 10^6^ PBMCs were stained with αA2-FITC (clone BB7.2; Serotec, Oxford, UK) for 30 min at 4 °C. For pMHCI tetramer staining, 1 × 10^6^ PBMCs were resuspended in phosphate-buffered saline and stained with LIVE/DEAD Fixable Violet (ViViD; Life Technologies) for 5 min at room temperature. After washing in phosphate-buffered saline, cells were stained with tetramer-PE (A2 wild type and variants thereof) at the indicated concentrations for 20 min at 37 °C. The following mouse anti-human monoclonal antibodies were then added for 20 min at 4 °C: αCD3-PerCP (clone SK7; BioLegend, San Diego, CA, USA); αCD4-FITC (clone VIT4; Miltenyi Biotec, Bergisch Gladbach, Germany); αCD8-APC (clone HIT8a; BD Pharmingen, San Diego, CA, USA); αCD14-Pacific Blue (clone HCD14; BioLegend); and αCD19-Pacific Blue (clone HIB19; BioLegend). Cells were washed twice in phosphate-buffered saline after staining and 5 × 10^4^ events per condition were acquired using a FACSCantoII flow cytometer (BD Biosciences, San Jose, CA, USA). Data were analyzed with FlowJo software version 10.6 (TreeStar Inc., Ashland, OR, USA).

### Macrophage inflammatory protein-1β enzyme-linked immunosorbent assay

Clonal CD8^+^ T-cells were incubated with C1R B cells expressing full-length A2 or variants thereof at different effector-to-target (E:T) ratios as indicated. Supernatants were collected after 18 h and assayed for macrophage inflammatory protein-1β by enzyme-linked immunosorbent assay according to the manufacturer's instructions (R&D Systems, Minneapolis, MN, USA).

### Chromium release assay

Target C1R B cells (1 × 10^6^) were loaded with ^51^Cr (30 μCi) for 1 h and plated in triplicate at 2 × 10^3^ cells per well in R10. Clonal CD8^+^ T cells were then applied at an E:T ratio of 5:1 in a final volume of 150 μl. Target cells incubated alone were used to calculate spontaneous release. Total release was measured via the addition of Triton X-100 (Sigma-Aldrich, St Louis, MO, USA). Supernatants were collected after 4, 6 or 18 h at 37 °C and mixed with OptiPhase Supermix Scintillation Cocktail (150 μl per well; PerkinElmer Life Sciences, Waltham, MA, USA). ^51^Cr content was measured using a MicroBeta Counter (PerkinElmer Life Sciences). Specific lysis (%) was calculated according to the following formula: (experimental release−spontaneous release/total release−spontaneous release) × 100.

### Surface plasmon resonance

Soluble TCRs and CD8αα were produced as described previously.^22, 46^ Binding analysis was performed using a BIAcore 3000 (GE Healthcare) equipped with a CM5 sensor chip. Between 200 and 400 response units of biotinylated pMHCI were immobilized to streptavidin, which was chemically linked to the chip surface. The pMHCI was injected at a slow flow rate (10 μl min^−1^) to ensure uniform distribution on the chip surface. Combined with the small amount of pMHCI bound to the chip surface, this reduced the likelihood of off-rate limiting mass transfer effects. Soluble MEL5 TCR and CD8αα were purified and concentrated to 100 and 150 μM, respectively, on the day of analysis to reduce the likelihood of aggregation affecting the results. For equilibrium analysis, eight serial dilutions of analyte were carefully prepared in triplicate for each sample and injected over the relevant sensor chips at 25 °C. Soluble MEL5 TCR or CD8αα were injected over the chip surface at a flow rate of 30 μl min^−1^. Results were analyzed using BIAevaluation 3.1 (GE Healthcare), Microsoft Excel (Microsoft, Redmond, WA, USA) and Origin 6.1 (OriginLab, Northampton, MA, USA). The equilibrium binding constant (*K*_D_) values were calculated using a nonlinear curve fit (*y*=[P1*x*]/[P2+*x*]).

### Statistical analysis

The dependence of nonspecific CD8^+^ T-cell staining intensity on tetramer concentration and the *K*_D_ of the pMHCI/CD8 interaction was assessed using the Friedman test for one-way effects and the Jonckheere–Terpstra test for the dependent variable increasing with the treatment variable.^47^

## Figures and Tables

**Table 1:** CD8-binding affinity measurements for the MHCI molecules used in this study

*Location of mutation*	*Description of mutation*	*pMHCI/CD8* K_*D*_ *(μM)*
MHCI α3 domain	A2 D227K/T228A	>10 000 (NDB)^a^
MHCI α3 domain	A2 A245V	498^a^
Wild type	No mutation	137±9.7^a^
MHCI α2 domain	A2 Q115E	98±14.5^a^
MHCI α3 domain	A2/K^b^ A245V	27±1
MHCI α3 domain	A2/K^b^	11^a^

Abbreviations: MHC1, major histocompatibility complex class I; NDB, no detectable binding; pMHC1, peptide-MHC1.

a Measurements reported previously for MHCI molecules refolded with wild type human β_2_m and the nonamer peptide LLFGYPVYV, an immunodominant epitope derived from the human T-cell lymphotropic virus type 1 Tax protein (residues 11–19).^17^
